# Transforming coffee from an empirical beverage to a targeted nutritional intervention: health effects of coffee's core functional components on chronic diseases

**DOI:** 10.3389/fnut.2025.1690881

**Published:** 2025-11-17

**Authors:** Rui Peng, Mengqi Lan, Yifei Zhang, Shishi Zhang, Beibei Yu, Xu Yang, Shiming Li, Zhenhua Liu, Wenyi Kang

**Affiliations:** 1 National R & D Center for Edible Fungus Processing Technology, Henan University, Kaifeng, China; 2 College of Agriculture, Henan University, Kaifeng, China; 3 Department of Food Science, Rutgers University, New Brunswick, NJ, United States; 4 Joint International Research Laboratory of Food & Medicine Resource Function, Kaifeng, China

**Keywords:** coffee, caffeine, chlorogenic acids, neuroprotective activity, diabetes mellitus, obesity, anti-inflammatory, antioxidant

## Abstract

As one of the most widely consumed beverages worldwide, coffee has garnered increasing scientific interest due to its potential health benefit in recent decades. Epidemiological studies have consistently shown that regular coffee consumption significantly reduces the incidence risks of various chronic diseases, including type 2 diabetes mellitus (T2DM), Alzheimer's disease (AD), cardiovascular disorders, and nephropathies. Pharmacological research further supports these findings, linking the protective effects of coffee to its complex composition of bioactive compounds. Coffee beans contain over 1,000 such compounds, with caffeine, trigonelline, chlorogenic acids (CGAs), cafestol, kahweol, and melanoidins constituting the core functional components. These phytochemicals act through multi-target, synergistic mechanisms that regulate neurological functions, metabolic homeostasis, and inflammatory pathways. This review systematically explores the major bioactive constituents of coffee, focusing on the molecular mechanisms underlying four key biological activities associated with chronic disease prevention: neuroprotection, anti-diabetic/anti-obesity effects, antioxidant activity, and anti-inflammatory properties. By elucidating these pharmacological pathways, we aim to establish a molecular theoretical foundation for repositioning coffee from an empirical beverage into a targeted nutritional intervention agent.

## 1 Introduction

Coffee, a dark beverage derived from the processed fruits of *Coffea* species in the Rubiaceae family, native to tropical regions, has long transcended its geographical origins. Since its first documented use as a stimulant in 15th-century Yemeni Sufi monasteries, coffee has evolved into a central element of global dietary culture (1). The global coffee industry continues to demonstrate strong growth, with the International Coffee Organization (ICO) reporting that, as of 2023, over 125 million people are employed across the coffee value chain. Annual production exceeds 10 million metric tons of coffee beans, with the global market value surpassing US$495.5 billion (1, 2).

Beyond its economic significance, the appeal of this dark brew lies in its multifaceted health effects, unveiled through interdisciplinary advances in nutritional epidemiology, molecular medicine, and systems biology. It also conforms to the theory of medicinal food homology and has nutritional and medicinal value (3, 4). Large-scale prospective cohort studies have consistently shown significant associations between coffee consumption and reduced risks of diabetes mellitus, neurodegenerative disorders, and cardiovascular diseases (5–8). Far from serving solely as a “caffeine vehicle,” coffee is a chemically complex beverage rich in bioactive compounds. Coffee beans contain polyphenols (e.g., CGAs), alkaloids (e.g., caffeine, trigonelline), diterpenes (e.g., cafestol), and trace minerals (magnesium, potassium) among others. Additionally, the roasting process produces Maillard reaction products such as melanoidins, collectively encompassing over 1,000 identified chemical constituents (5, 6, 9).

Over the past decade, research paradigms have shifted from macro-level association analyses to investigations of molecular mechanisms: **Caffeine** (1,3,7-trimethylxanthine), a central nervous system (CNS) stimulant, enhances cognitive function and reduces Parkinson's disease (PD) risk through adenosine A_2A_ receptor (A_2A_R) antagonism, though its dose-dependent effects may induce anxiety and disrupt calcium (Ca^2+^) homeostasis at high intake levels (6). **Chlorogenic acid** (5-*O*-caffeoylquinic acid, 5-CQA), the predominant polyphenol in coffee, activates the nuclear factor erythroid 2-related factor 2 (Nrf2) pathway to suppress oxidative stress and inhibits α-glucosidase to modulate postprandial glycemia. **Diterpenes** such as cafestol exhibit dual effects—potentially elevating the levels of low-density lipoprotein cholesterol (LDL-C) while inducing glutathione-S-transferase (GST) for anticancer activity (7, 8). **Melanoidins** demonstrate metal-chelating properties, however, their concomitant formation with acrylamide (a Group 2A carcinogen per IARC classification) warrants caution (7).

The diverse chemical constituents of coffee collectively orchestrate health outcomes through intricate synergistic, antagonistic, or cascading interactions across multiple molecular targets, regulating oxidative stress, inflammatory responses, metabolic pathways, and neuroprotective mechanisms.

Current pharmacological research on coffee remains fragmented, often emphasizing isolated constituents while lacking a systematic understanding of multicomponent synergies. This review integrates molecular evidence linking key coffee bioactives to four primary health domains: neuroprotection, metabolic regulation, antioxidant activity, and anti-inflammatory effects. By mapping the target networks of CGAs, caffeine, and related compounds, we elucidate the biological basis of coffee's “multi-target regulation.” This integrated perspective advances a more holistic understanding of coffee as a functional food and provides a theoretical fondation for its health-promoting properties.

## 2 Principle bioactive compounds in coffee

Commercial coffee cultivation primarily involves three species: *Coffea arabica* L. (Arabica), *Coffea canephora* Pierre ex A. Froehner (Robusta), and *Coffea liberica* Hiern (Liberica). Of these, Arabica accounts for ~70% of the global market share, favored for its low bitterness and complex aromatic profile (5). Coffee processing systems encompass traditional methods such as dry (natural), wet (washed), and honey (pulped natural) processing. Brewing techniques are equally diverse, including espresso, French press, American drip, Turkish preparation, moka pot, and instant coffee preparations (5). The health impacts of coffee are closely tied to its complex chemical composition, which exhibits dynamic variations influenced by genetic differences, roasting degrees, and brewing methods.

Raw (unroasted) coffee beans are primarily composed of carbohydrates (59–61%), lipids (8–18%), and proteins (9–16%), supplemented by nitrogenous compounds (1–6%), minerals (4%), and acid-ester substances (6–15%) (10). Roasting induces significant compositional changes driven by Maillard reactions and pyrolysis. Carbohydrates contents decrease to 38–42%, lipids rise to 10–15%, proteins adjust to 7.5–10%, while nitrogenous compounds diminish to 1–2%. A notable outcome of roasting is the formation of melanoidins—characteristic Maillard reaction products—which comprise about 25% of the roasted bean mass. Meanwhile, mineral content stabilizes at 3.7–5%, and acid-ester compounds remain constant at 6% (11). This gradient transformation in chemical composition directly reflects the complex biochemical changes during thermal processing.

The bioactive constituents of coffee are broadly classified into four major categories: **Alkaloids** (e.g., caffeine, trigonelline); **Polyphenols** (e.g., CGAs); **Diterpenes** (e.g., cafestol); **Maillard reaction products** (e.g., melanoidins). These compounds interact through complex synergistic and antagonistic mechanisms, forming multidimensional regulatory networks that underpin coffee's diverse health effects-ranging from neuroprotection to metabolic homeostasis (6, 8, 9).

### 2.1 Alkaloids

**Figure 1:**
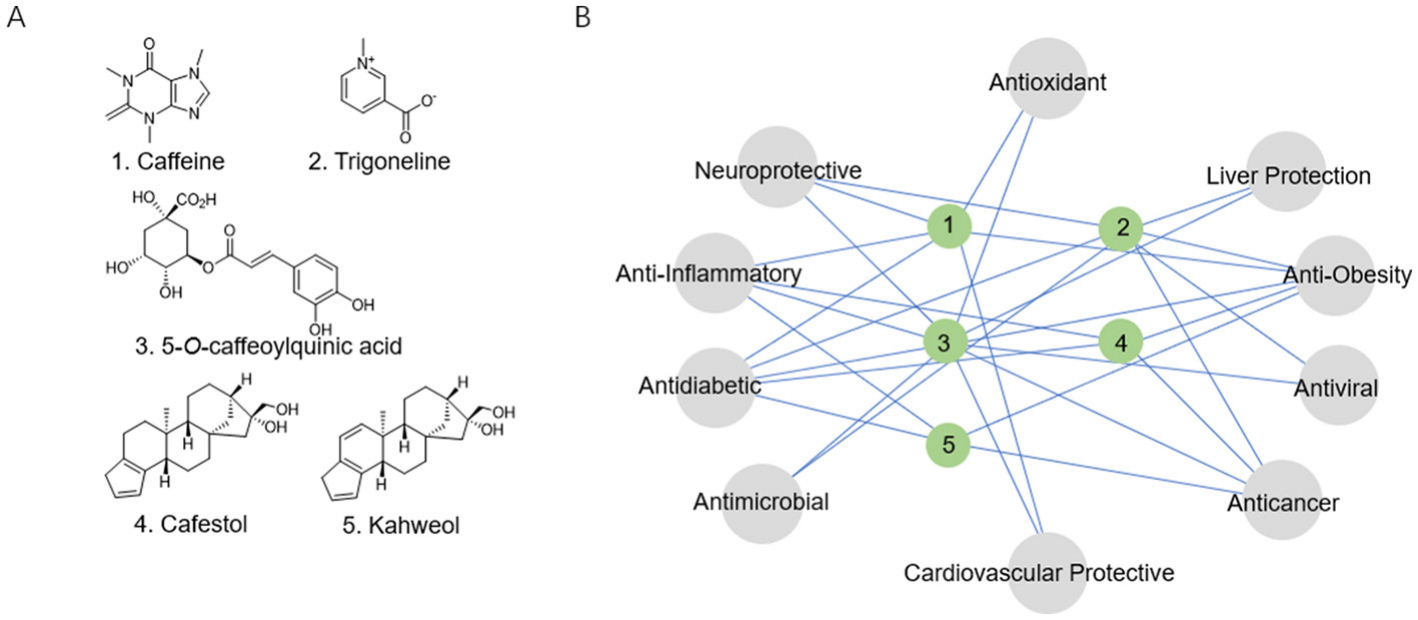
**(A)** Structural formulas of the coffee's core functional components. **(B)** The relationship network between the coffee's core functional components and biological activity.

Alkaloids were among the first bioactive constituents of coffee to be studied, with caffeine recognized as the primary compound due to its rapid physiological responses and dose-sensitive properties (12). Caffeine (Figure 1A), a methylxanthine derivative, possesses a planar, rigid molecular structural configuration that facilitates efficient transmembrane diffusion, allowing for swift accumulation in CNS (12). Significant interspecies variations exist in caffeine content: *C. arabica* contains 1.2–1.5% by dry weight, whereas *C. canephora* (Robusta) contains significantly more, ranging from 2.2 to 2.7% (13). Caffeine remains highly stable during thermal processing and is primarily metabolized in the liver through P450 1A2 (CYP1A2). Its metabolic clearance is modulated by genetic polymorphisms in cytochrome P450 enzymes (14).

Caffeine exerts its physiological functions primarily through mechanisms: competitive antagonism of adenosine A_1_/A_2A_ receptors; and specific inhibition of phosphodiesterase 4/5 (PDE4/5) activity, leading to elevated intracellular cyclic adenosine monophosphate/cyclic guanosine monophosphate (cAMP/cGMP) levels, which in turn enhance Ca^2+^ release, caffeine behaves as a low-affinity benzodiazepine-site antagonist at γ-aminobutyric acid type A (GABA_A) receptors. While the latter interaction contributes to the vigilance-promoting properties of the methylxanthine, it may also underlie its anxiogenic potential and the observed reduction in seizure threshold (15, 16). Dose-response studies reveal beneficial that low to moderate caffeine intake—typically 50–200 mg/day (equivalent to 1–2 cups of coffee) or 200–400 mg/day (3–5 cups)—is associated with various health benefits. However, excessive intake (>500 mg/day, or more than six cups) may lead to adverse effects including heightened anxiety and sleep disturbances, a single serving of coffee, providing approximately 100 mg of caffeine, yields mean plasma caffeine concentrations of 10 and 19 μmol/L in male and female individuals, respectively (15, 17–19). Caffeine has been demonstrated to exhibit a range of pharmacological activities, including neuroprotective, anti-obesity, anti-inflammatory, and anti-diabetic effects (Figure 1B) (15, 17).

Trigonelline (Figure 1A), the second most abundant pyridine alkaloid in coffee, constitutes 1–3% of the dry mass of raw beans (20). Its distinctive chemical properties and metabolic transformations contribute to a range of health benefits, particularly in the management of diabetes and the prevention of neurodegenerative diseases (Figure 1B) (20, 21).

### 2.2 Polyphenols

Polyphenols constitute the most abundant antioxidants in coffee. CGAs, the predominant polyphenolic compounds, serve as core contributors to coffee's antioxidant and metabolic regulatory properties. CGAs are hydroxycinnamic acid derivatives (molecular formula C16H18O9) formed via ester bonds between caffeic and quinic acids (22). Their catechol moieties impart strong antioxidant properties, with 5-CQA (Figure 1A) comprising over >75% of total CGAs (23). Substantial varietal differences are observed: *C. arabica* beans contain 5-8% CGAs by dry weight, while *C. canephora* (Robusta) ranges from 7 to 10%. Thermal processing significantly degrades CGAs—Light roasting (180°C) retains 60–70%, Medium roasting (210°C) preserves 30–40%, Dark roasting (240°C) leaves only 10–15%. Degradation products include caffeic acid, quinic acid, and chlorogenic acid lactones, the latter contributing to coffee's bitterness (24). Despite low oral bioavailability (3–5%), CGAs exhibit multidimensional bioactivities encompassing antioxidant effects, glycemic regulation, anti-inflammatory actions, and neuroprotection (Figure 1B) (22, 24). A typical serving of coffee contains approximately 27.33–121.25 mg of chlorogenic acids, with 5-CQA accounting for 36–40% of this content. Following consumption, approximately one-third of the ingested 5-CQA enters the systemic circulation, reaching peak plasma concentrations within 0.5–1.5 h to exert its pharmacological activity (16, 25).

### 2.3 Diterpenes

Cafestol and kahweol (Figure 1A), unique furan diterpenes predominantly concentrated in coffee oils, constitute 19–22% of the lipid fraction in coffee beans (26). Their physiological effects are highly dependent on brewing method: paper-filtered preparations remove over 85% of these diterpenoids, whereas unfiltered brewing techniques such as French press and Turkish coffee retain their full content (6–12 mg per cup) (27). These compounds exhibit paradoxical biological activities. While they may raise low-density lipoprotein (LDL) cholesterol levels and potentially elevating cardiovascular disease risk, they also confer beneficial effects, including anti-inflammatory, hepatoprotective, and anti-carcinogenic properties (Figure 1B) (26, 27).

### 2.4 Maillard reaction products

The Maillard reaction, a key process in coffee roasting, generates a complex array of compounds, among which melanoidins are prominent characteristic high-molecular-weight polymers (5–100 kDa) formed through condensation reactions between reducing sugars and amino acids at 140–200°C. These melanoidins exhibit furan and pyrrole ring-enriched molecular structures, endowing them with transition metal ion chelation capacity and lipid peroxidation inhibitory activity (28). However, this reaction also produces potentially harmful substances such as acrylamide, a Group 2A carcinogen (IARC classification) derived from the Strecker degradation of asparagine and reducing sugars at temperatures exceeding 120 °C. Dark-roasted coffee may contain acrylamide levels up to 10 μg/kg, raising concerns due to its neurotoxic and carcinogenic concerns (28, 29).

## 3 Therapeutic potential and pharmacological mechanisms of coffee

### 3.1 Neuroprotective effects

With the rapidly global rise in the elderly population, neurodegenerative diseases have emerged as major public health challenges. Developing interventions to delay neuronal damage and preserve cognitive function is crucial for preventing and managing conditions such as AD and PD. AD, the most prevalent neurodegenerative disorder affecting over 24 million patients worldwide, is characterized by progressive cognitive decline encompassing memory loss, language deficits, and impaired executive dysfunction. Its pathogenesis involves extracellular β-amyloid (Aβ) plaque deposition and intraneuronal neurofibrillary tangles (NFTs) formed by hyperphosphorylated Tau protein (pTau), ultimately leading to neuronal toxicity and death (30). PD, the second most common neurodegenerative disease, exhibits age-related increases in prevalence and is characterized primarily by the selective degeneration of dopaminergic neuron in the substantia nigra pars compacta (31). In both AD and PD, mitochondrial dysfunction, reactive oxygen species (ROS)-mediated macromolecular damage, and microglial overactivation—leanding to the release of pro-inflammatory cytokines [e.g., tumor necrosis factor-α (TNF-α), interleukin-1β (IL-1β)] collectively exacerbate neurodegeneration in both AD and PD (32).

Recent studies demonstrate an inverse correlation between coffee consumption and the risk of neurodegenerative diseases. A 2022 multinational meta-analysis of 6,121 subjects found that moderate daily intake of 1–4 cups of coffee was associated with a reduced incidence of Alzheimer's disease, whereas excessive consumption (>4 cups/day) may yield counterproductive effects (33). Similarly, a large-scale cohort study involving 389,505 participants identified 2.5 cups per day as the optimal protective threshold against AD (34). Furthermore, a 126-month longitudinal study of 227 cognitively normal elder adults observed slower cerebral Aβ deposition rates in high coffee intake groups, suggesting that coffee may potentially attenuate cognitive decline through Aβ pathology suppression (35). A 9-year follow-up study of 1,696 AD-related dementia (ADRD), 1,093 PD, and 419 neurodegenerative disease death cases revealed that coffee consumption was significantly associated with reduced risks of ADRD and PD. Notably, a comparison between caffeinated and decaffeinated coffee consumers highlighted that specifically caffeinated coffee was linked to a lower risk of neurodegenerative diseases and associated mortality, underscoring the critical role of caffeine (36). Regarding caffeine, a Meta-Analysis indicated that it not only reduces the risk of Parkinson's disease in healthy individuals but also ameliorates disease progression in patients with established Parkinson's (37). Similarly, a separate double-blind, randomized controlled trial further confirmed the beneficial effect of caffeine on motor function in PD patients (38).

The neuroprotective effects of coffee arise from the synergistic actions of its bioactive components. Caffeine acts as an adenosine A_2A_ receptor antagonist, modulating neurotransmission and promoting neuronal survival, while CGAs mitigate neural damage through potent antioxidant and anti-inflammatory mechanisms (32).

#### 3.1.1 Caffeine

**Figure 2:**
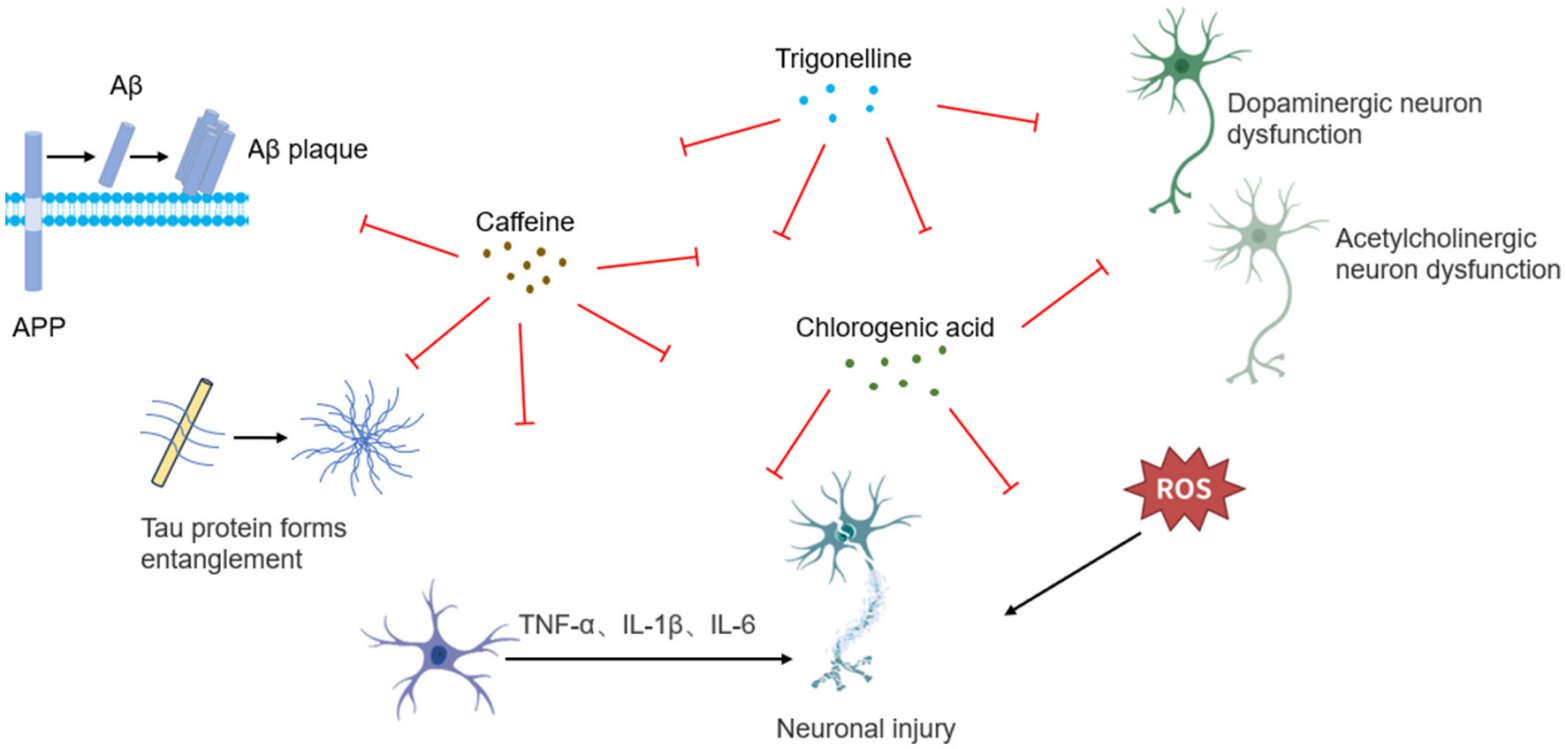
Schematic illustration of neuroprotective mechanisms underlying coffee's core functional components.

Caffeine exerts neuroprotective effects through the synergistic modulation of multiple pathways, including adenosine receptor antagonism, neuroinflammatory suppression, oxidative stress reduction, mitochondrial function enhancement, and synaptic plasticity regulation (Figure 2). In streptozotocin (STZ)-induced AD rat models, caffeine significantly decreased hippocampal oxidative stress markers, inhibited Aβ plaque deposition and tau hyperphosphorylation, while enhancing neural progenitor cell proliferation and improving spatial memory function (39). Rotenone-induced PD rat models, caffeine was shown to restore tyrosine hydroxylase (TH) expression and cytoplasmic NRF2 levels, thereby reversing acetylcholine esterase (AChE) depletion (40).

In 1-methyl-4-phenyl-1,2,3,6-tetrahydropyridine (MPTP)-induced PD mouse models, caffeine administration reversed striatal dopamine (DA) depletion, improved motor coordination, and reduced the number of MPTP-induced glial fibrillary acidic protein (GFAP)-positive cells. This intervention also restored mitochondrial complex I activity inhibition, decreased ROS generation, and elevated glutathione (GSH) levels, highlighting its antioxidative, anti-inflammatory, and dopaminergic neuroprotective mechanisms (41). In aluminum chloride (AlCl_3_)-induced neurotoxicity models, it is revealed that caffeine reduced cortical lipid peroxidation and nitric oxide (NO) levels, restored GSH homeostasis, inhibited aberrant AChE and Na+/K+-ATPase activation, and downregulated pro-inflammatory cytokine TNF-α (42). In chronic hypoxia-induced cognitive impairment mouse models, caffeine modulated striatal DA metabolic homeostasis via adenosine A_2A_ receptor blockade and TH expression upregulation in dopaminergic neurons (42).

#### 3.1.2 Chlorogenic acid

The neuroprotective effects of CGA stem from its multidimensional regulatory capabilities, encompassing free radical scavenging, neuroinflammatory suppression, apoptotic pathway inhibition, and autophagy promotion, collectively maintaining neuronal homeostasis (Figure 2). In pentylenetetrazole (PTZ)-induced epileptic mouse models, CGA significantly improved behavioral abnormalities, enhanced AChE activity, and increased levels of brain-derived neurotrophic factor (BDNF), as well as monoamines including DA, norepinephrine (NE), and serotonin (5-HT). Its antioxidant effects were evidenced by elevated GSH levels, upregulation of Nrf2 gene expression, and reductions in malondialdehyde (MDA) and NO concentrations. Anti-inflammatory mechanisms of CGA involved suppression of pro-inflammatory cytokines—IL-1β, IL-6, and TNF-α, alongside inhibition of nuclear factor kappa-light-chain-enhancer of activated B cells (NF-κB) nuclear translocation (p65), and downregulation of inducible nitric oxide synthase (iNOS) expression. Furthermore, CGA significantly reduced pro-apoptotic protein Bax expression and caspase-3 activity, demonstrating multi-pathway synergistic protection (43). In lithium-pilocarpine-induced epilepsy models in rats, CGA similarly conferred neuroprotective effects by alleviating hippocampal oxidative stress and reducing neuronal apoptosis (44). In kainic acid-induced rat models, CGA alleviated excitatory neurotoxicity through glutamate homeostasis maintenance, AMP-activated protein kinase (AMPK)/sirtuin 1 (SIRT1)/ peroxisome proliferator-activated receptor gamma coactivator 1-alpha (PGC-1α)-mediated mitochondrial biogenesis activation, and PTEN-induced putative kinase 1 (PINK1)/Parkin-dependent mitophagy enhancement (45). In 6-hydroxydopamine (6-OHDA)-induced neuronal injury models, CGA decreased the levels of ROS, suppressed excessive extracellular signal-regulated kinases 1/2 (Erk1/2) phosphorylation, and prevented Akt signaling inactivation, thereby attenuating dopaminergic neuron apoptosis. In A53T α-synuclein transgenic mice, CGA further alleviated PD pathology through modulation of the Akt/Erk1/2 signaling cascade (46). In MPTP-induced zebrafish models, CGA demonstrated the ability to reverse dopaminergic neuron loss, improve motor dysfunction, and reduce α-synuclein aggregation (47). Complementary studies in MPTP-induced PD mice confirmed its neuroprotective effects through mitochondrial dysfunction amelioration, apoptotic signaling inhibition, and Akt/Erk1/2/ glycogen synthase kinase 3 beta (GSK3β) phosphorylation network regulation (48). In neonatal rat models of hypoxic-ischemic brain injury (HIE), CGA promoted Nrf2 nuclear translocation, suppressed NF-κB activation, and upregulated heme oxygenase-1 (HO-1), silent information regulator 1 (Sirt1), and IκBα. Subsequent *in vitro* studies using cortical neuron further validated its SIRT1-mediated regulation of the NRF2/NF-κB signaling axis in preserving neuronal viability (49). Additional models consistently corroborated CGA's broad neuroprotective efficac.

#### 3.1.3 Trigonelline

Evidence from *in vitro* studies, animal models, and molecular docking analyses suggests that trigonelline holds therapeutic potential for neurodegenerative disorders including AD, PD, and depression through mechanisms involving oxidative stress mitigation, neuroinflammatory suppression, and AChE inhibition (Figure 2) (50–52). In senescence-accelerated mouse prone 8 (SAMP8) models, trigonelline significantly reduced hippocampal pro-inflammatory cytokines TNF-α and IL-6, while increasing concentrations of neurotransmitters including DA, NE, and serotonin, thereby ameliorating cognitive dysfunction (50). In models of oxygen-glucose deprivation/reperfusion (OGD/R)-induced hippocampal neuronal injury, trigonelline conferred neuroprotection via modulating the phosphoinositide 3-kinase (PI3K)/Akt pathway, exerting antioxidative, anti-inflammatory, and anti-apoptotic effects to preserve neuronal structural integrity (53). Investigations using 5XFAD transgenic AD models carrying APP/PS1 mutations further demonstrated trigonelline's ability to activate creatine kinase B-type (CK-B), promote axonal regeneration, reverse β-Aβ-induced axonal degeneration, and enhance memory function (51). Additionally, in lipopolysaccharide (LPS)-induced cognitive impairment models, trigonelline alleviated hippocampal oxidative stress and inflammation by suppressing the NF-κB/ Toll-like receptor 4 (TLR4) signaling pathway and modulating AChE activity, ultimately improving cognitive performance (54).

### 3.2 Antidiabetic and anti-obesity effects

Diabetes mellitus (DM) and obesity are major global metabolic disorders that pose significant public health challenges due to their high prevalence and pathophysiological synergism. According to the World Health Organization (WHO), over 10% of adults worldwide suffer from diabetes (predominantly T2DM), while the obese population exceeds 650 million. These metabolic dysregulations frequently coexist and exert marked synergistic pathological effects. Obesity, a key risk factor for T2DM, contributes to metabolic dysfunction predominantly through adipose tissue dysregulation. Dysfunctional white adipose tissue (WAT) expansion leads to leptin resistance and decreased adiponectin secretion, while released free fatty acids (FFAs) induce insulin resistance (IR) in peripheral tissues including skeletal muscle and liver. Concurrently, chronic low-grade inflammation triggered by adipose tissue macrophage infiltration impairs insulin signaling pathways (e.g., insulin receptor substrate (IRS)-PI3K-AKT cascade) via the release of pro-inflammatory cytokines such as TNF-α and IL-6, thereby exacerbating glucolipid metabolic disturbances. Studies further confirm pathological links between obesity-associated mitochondrial dysfunction, endoplasmic reticulum stress, gut microbiota dysbiosis, and pancreatic β-cell decompensation in T2DM progression (55). The cross-sectional study of 2,556 adults revealed that coffee consumption was associated with more favorable body composition parameters, including lower body mass index (BMI) and waist girth, as well as reduced systemic inflammation, indicated by lower high-sensitivity C-reactive protein (hs-CRP) levels (56). In a 3-year follow-up study of a cohort characterized by advanced age, high cardiovascular risk, elevated obesity rates, dyslipidemia, and a high prevalence of T2DM, the new initiation of moderate caffeinated coffee consumption was associated with reductions in both total body fat and visceral adipose tissue (57). A randomized, controlled, crossover study revealed that lightly roasted coffee led to a greater reduction in body fat percentage in participants compared to roasted coffee, an effect attributable to its higher content of CGA and caffeine (58). Furthermore, additional studies have demonstrated that coffee consumption can reduce daily energy intake and increase energy expenditure (59, 60). Notably, evidence from cohort studies and Mendelian randomization analyses reveals inverse correlations between coffee consumption and T2DM/obesity risks, with coffee extracts exhibiting anti-obesity bioactivity (55, 61, 62).

#### 3.2.1 Caffeine

**Figure 3:**
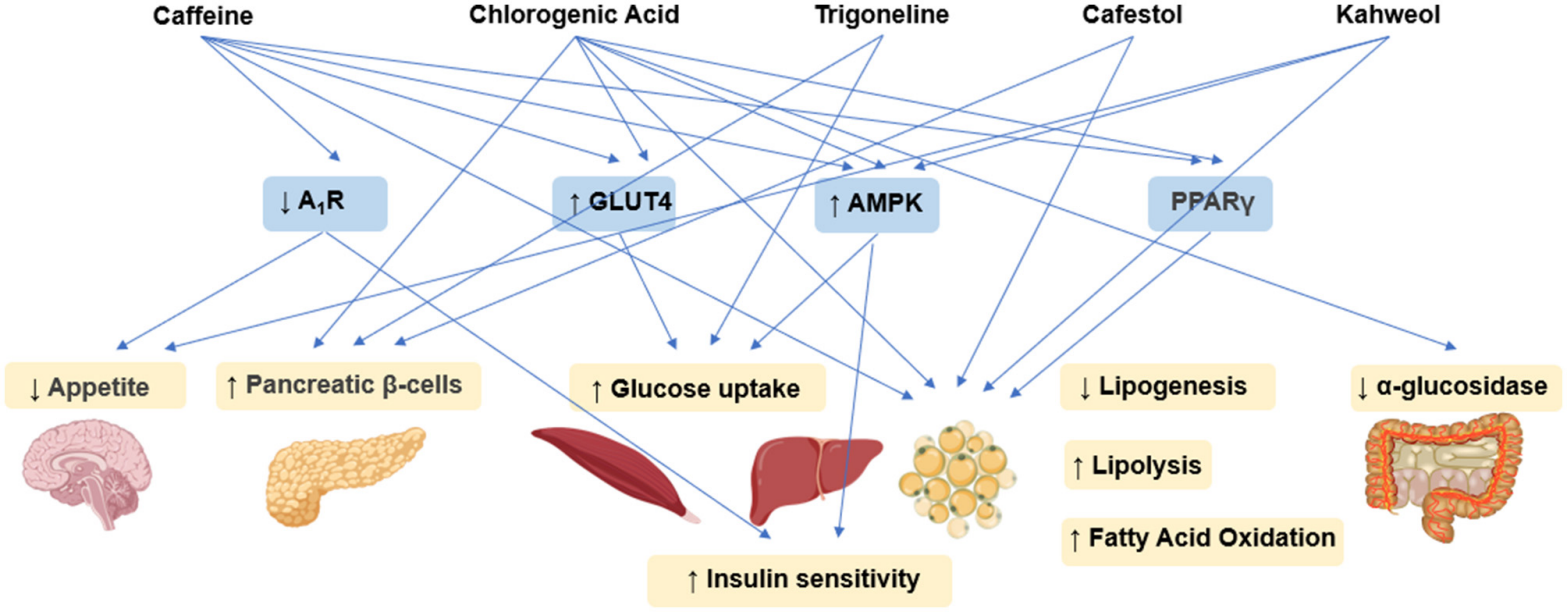
Schematic illustration of antidiabetic and anti-obesity effects mechanisms underlying coffee's core functional components.

Caffeine exerts anti-obesity and anti-diabetic effects through coordinated central-peripheral synergistic regulation mechanisms. It integrates energy metabolism and glucolipid homeostasis networks via hypothalamic appetite suppression, adipose tissue lipolysis activation, and insulin sensitization (Figure 3) (63). In diabetic Wistar rat models, caffeine intervention significantly reduced fasting blood glucose (FBG) levels and improved glucose tolerance through upregulating skeletal muscle glucose transporter 4 (GLUT4) expression, activating AMP-activated protein kinase (AMPK) to enhance peripheral glucose uptake, and antagonizing adenosine A_1_ receptor (A_1R_) to improve insulin sensitivity (64). In diet-induced obesity (DIO) mouse models, caffeine exerted dual regulatory effects through A_1R_-mediated suppression of oxytocin neurons in the paraventricular nucleus (PVN), leading to reducing food intake, increased energy expenditure, and ultimate body weight reduction. These effects were accompanied by improved plasma triglyceride (TG) levels and glucose tolerance (65). Combined studies using 3T3-L1 adipocytes and C57BL/6J mice revealed caffeine's metabolic regulatory actions through multi-target mechanisms by suppressing adipogenic proteins [FASN, p-acetyl-CoA carboxylase (pACC)] to reduce lipid droplet accumulation, activating the AMPK/SIRT1 axis to enhance autophagy, and promoting white adipose browning and mitochondrial biogenesis. These mechanisms collectively contributed to attenuated weight gain, improved insulin sensitivity, and optimized lipid profiles, supporting caffeine's metabolic benefits (66). Molecular further revealed that caffeine inhibited adipogenic enzyme activities (GPDH, FAS) while activating lipolytic enzymes [hormone-sensitive lipase (HSL)] in 3T3-L1 cells. This process involved downregulation of adipogenic transcription factors [PPAR-γ2, CCAAT/enhancer-binding protein alpha (C/EBPα)] mRNA expression and upregulation of fatty acid oxidation [ACO, carnitine acyltransferase (CAT)] and lipolysis [adipose triglyceride lipase (ATGL, HSL)] genes, suggesting AMPK-mediated reprogramming of lipid metabolism (67). Animal studies further confirmed caffeine's anti-obesity effects through modulation of hepatic AMPKα-liver X receptor alpha (LXRα)/sterol regulatory element-binding protein 1c (SREBP-1c) signaling pathway (68).

#### 3.2.2 Chlorogenic acid

CGA ameliorates glucolipid metabolic disorders through multi-target synergistic mechanisms (Figure 3). Its anti-diabetic and anti-obesity effects primarily contributed to α-glucosidase inhibition to delay carbohydrate absorption, AMPK/PPARγ pathway activation to enhance insulin sensitivity, and bidirectional regulation of lipolysis/adipogenesis-related genes for metabolic homeostasis. In high-fat diet/streptozotocin (STZ)-induced T2DM rat models, CGA intervention significantly reduced the levels of FBG, serum total cholesterol (TC), and TGs, while emhancing insulin expression in pancreatic β-cells. Transcriptomics and network pharmacology analyses identified modulation of the advanced glycation end products (AGE)-receptor for AGE (RAGE) signaling pathway as a key mechanism underlying its hypoglycemic effects (69). In obesity animal models, CGA activated AMPK signaling, thereby promoting lipolysis and fatty acid β-oxidation while suppressing key lipogenic enzyme activities (70). Experiments using 3T3-L1 preadipocytes further confirmed CGA enhances cell viability, reduces lactate dehydrogenase (LDH) release as an oxidative damage marker, and inhibits adipocyte differentiation through multiple mechanisms. These include: decreasing ROS generation while elevating superoxide dismutase (SOD), GSH, CAT levels; suppressing pro-inflammatory cytokines (TNF-α, IL-6, IL-1β) with concomitant IL-10 upregulation; and reducing lipid droplet size and TG content via downregulation of adipogenic genes (PPARγ2, SREBP1, C/EBPα) and modulation of lipid metabolism genes (FAS, ACC, AMPK, ACO, HSL) (71). Additionally, *In vitro* enzyme kinetics revealed that CGA's α-glucosidase inhibitory pattern exhibits concentration-dependent mode-switching—competitive inhibition at low concentrations transitioning to noncompetitive inhibition at higher concentrations, demonstrating mixed-type inhibition characteristics (72). In *db/db* diabetic mice, CGA markedly decreased body weight and blood glucose by upregulating fatty acid oxidation genes (*CPT1*α*, ACOX1*) and lipolytic genes (*ATGL, HSL*), while downregulating lipogenic genes (*MGAT1, DGAT1/2*) and lipid transporter genes (*CD36, FATP4*), therby effectively alleviating hepatic lipid accumulation (73). Experiments using C3H10T1/2 cells demonstrated that CGA activates AMPK signaling, thereby promoting glucose the expressin of transporter GLUT2 and phosphofructokinase (PFK), enhancing glucose uptake and glycolytic efficiency, inducing mitochondrial biogenesis, ultimately upregulating uncoupling protein 1 (UCP1)-mediated thermogenesis (74).

#### 3.2.3 Trigonelline

Trigonelline, a characteristic alkaloid in coffee, has demonstrated anti-diabetic potential through multi-dimensional mechanistic studies (Figure 3) (75). In HFD-STZ-induced diabetic rat models, Trigonelline synergistically supports pancreatic β-cell function by modulating glucose transporters and key glycolytic enzyme activities, significantly improving insulin sensitivity and reducing blood glucose and lipid profiles (76). Further studies in the same models revealed that trigonelline effectively suppresses β-cell apoptosis through the downregulation of the pro-apoptotic factor caspase-3 and enhancement of antioxidant enzyme activities. These anti-diabetic effects appear to be closely associated with inflammatory response modulation (77). Notably, trigonelline ameliorates dysregulated lipid absorption by activating key intestinal lipid-metabolizing enzymes, manifesting as reduced serum TGs and total TC levels alongside elevated high-density lipoprotein cholesterol (HDL-C) (78). In studies on complications associated with T2DM, trigonelline has been shown to significantly slow the progression of diabetic nephropathy and enhance insulin sensitivity, primarily through activation of the PPAR-γ signaling pathway (79).

#### 3.2.4 Cafestol and Kahweol

Cafestol and kahweol, two characteristic diterpenoids found in coffee, exhibit dual regulatory effects on glucolipid metabolism (Figure 3) (80). Studies demonstrate that kahweol inhibits lipid accumulation in 3T3-L1 adipocytes and enhances glucose uptake through the activation of AMPK signaling pathway (81), while cafestol exerts hypoglycemic effects by stimulating insulin secretion in pancreatic β-cells and augmenting glucose transport in skeletal muscle cells (82). In *Caenorhabditis elegans* models, cafestol increases energy expenditure by activating key fat-oxidizing enzymes via the DAF-12-dependent pathway (83). In streptozotocin (STZ)-induced diabetic rat models, kahweol has been shown to enhancere insulin secretion in INS-1 cells through NF-κB inflammatory pathway modulation and p-AKT/B-cell lymphoma 2 (Bcl-2) apoptosis signaling regulation (84). Molecular investigations further demonstrate that kahweol suppresses 3T3-L1 preadipocyte differentiation by downregulating the core adipogenic transcription factor PPARγ (85).

### 3.3 Anti-inflammatory effects

Inflammation, an evolutionarily conserved defense mechanism in response to pathogen invasion, tissue injury, or endogenous danger signals, is characterized by pathological processes such as immune cell activation, the release of pro-inflammatory mediators, and changes in microvascular permeability (86). Clinically, inflammation is categorized into acute and chronic forms. Acute inflammation features neutrophil infiltration and cardinal clinical signs - *rubor* (redness), tumor (swelling), calor (heat), dolor (pain), mediated through precisely orchestrated molecular cascades involving NF-κB and mitogen-activated protein kinase (MAPK) signaling pathways. Chronic inflammation manifests as prolonged monocyte/macrophage activation, aberrant secretion of pro-inflammatory cytokines such as TNF-α, IL-1β, IL-6, and tissue fibrotic remodeling. It plays a critical role in the pathogenesis of metabolic syndrome, neurodegenerative disorders, and malignancies (86). A meta-analysis of 11 cross-sectional studies involving 66,691 participants revealed that coffee consumption is associated with reduced C-reactive protein (CRP) levels (87). Furthermore, two additional studies further support an inverse association between coffee consumption and CRP levels (36, 88). A multi-omics analysis following coffee consumption revealed a significant downregulation of pro-inflammatory genes and concurrent upregulation of anti-inflammatory genes. This coordinated shift led to reduced serum levels of inflammatory cytokines, suggesting a potential anti-inflammatory effect of coffee (89).

#### 3.3.1 Caffeine

**Figure 4:**
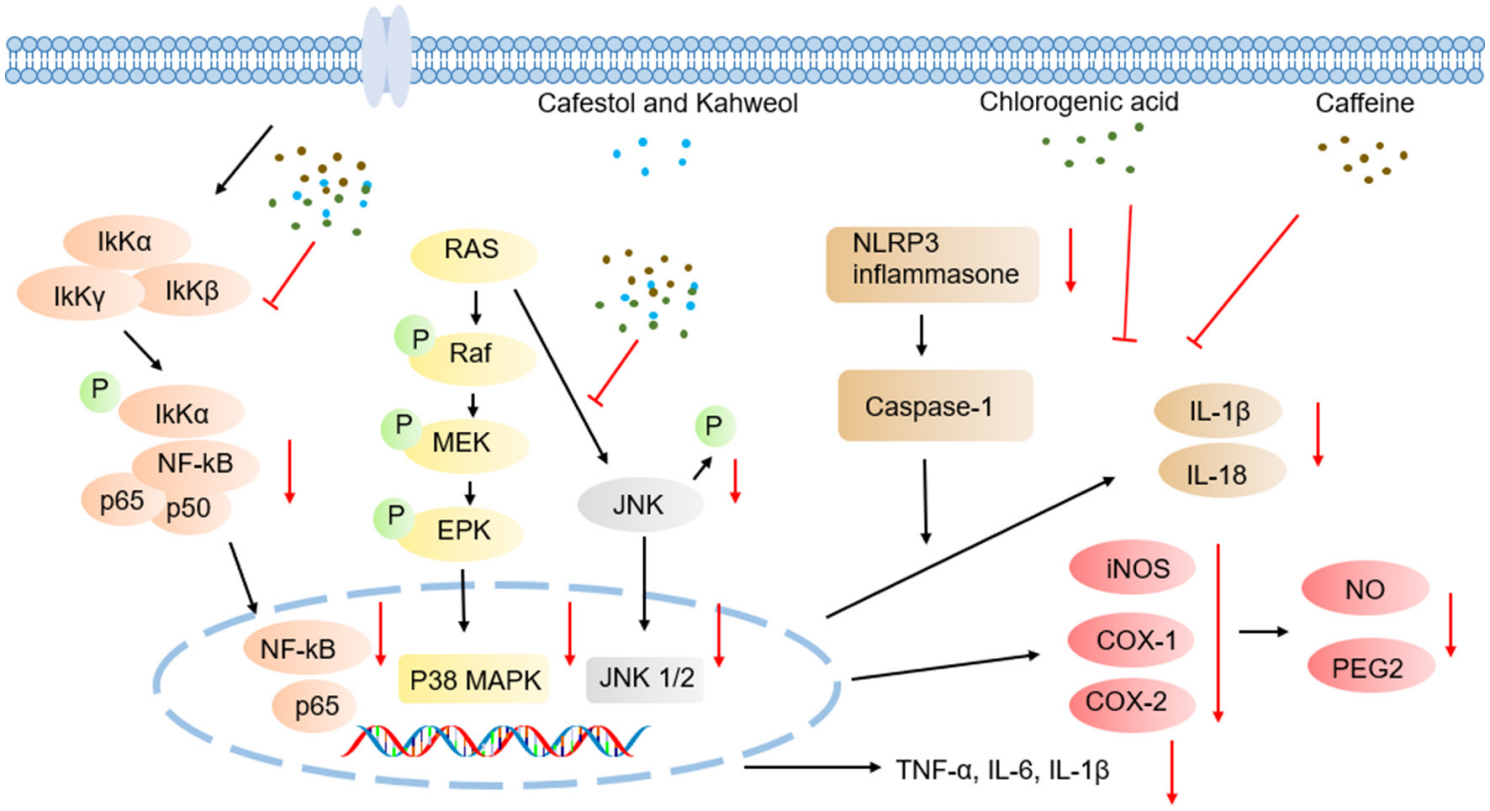
Schematic illustration of anti-inflammatory effects mechanisms underlying coffee's core functional components.

Caffeine modulates inflammatory cascades through multi-target synergistic mechanisms, including inhibition of NF-κB and MAPK signaling pathways, blockade of NLRP3 inflammasome assembly, and downregulation of pro-inflammatory cytokines such as TNF-α, IL-6, and IL-1β (Figure 4) (86, 90). In lipopolysaccharide (LPS)-induced neuroinflammatory models using male C57BL/6J mice, caffeine intervention significantly elevated BDNF levels, while concurrently suppressing pro-inflammatory cytokines including TNF-α, IL-6, IL-1β, and interferon-gamma (IFN-γ), and reducing lipid peroxidation (91). Further studies in neuroinflammatory-depressive comorbidity rat models have demonstrated caffeine's ability to regulate LPS-induced cortical p-AKT and NF-κB expression, indicating anti-inflammatory effects via AKT phosphorylation/NF-κB signaling axis modulation (92). Complementary experiments employing human retinal pigment epithelial cells (ARPE-19) and C57BL/6J mice confirmed that caffeine effectively inhibits LPS-triggered inflammation by reducing mRNA expression of TNF-α, IL-6, and IL-1β, inhibiting p-NF-κB p65 nuclear translocation, restoring monolayer integrity, and upregulating BDNF levels, revealing its potent protective anti-inflammatory role in retinal (93). In models of non-alcoholic steatohepatitis (NASH), caffeine ameliorated hepatic inflammation by downregulating TLR4/MAPK/NF-κB signaling and suppressing NLRP3 inflammasome activation (94).

#### 3.3.2 Chlorogenic acid

CGA exerts anti-inflammatory effects through multi-target synergistic mechanisms, including inhibition of NF-κB and MAPK signaling pathway activation, blockade of NLRP3 inflammasome assembly, and downregulation of pro-inflammatory cytokines such as TNF-α, IL-6, and IL-1β (Figure 4). Additionally, CGA activates the Nrf2/ARE antioxidant pathway to alleviate oxidative stress-associated inflammation (95–100). In a cecal ligation and puncture (CLP)-induced septic acute liver injury (SALI) mouse model, CGA dose-dependently ameliorated hepatic tissue damage. qPCR and Western blot analyses confirmed its significant inhibition of pro-inflammatory cytokines in TNF-α and IL-1β expression, along with downregulation of TLR4 and NF-κB, indicating that its anti-inflammatory effects are closely linked to modulation of TLR4/NF-κB signaling axis (101). In LPS-stimulated RAW264.7 macrophage models, CGA concurrently inhibited inflammatory mediator secretion and signaling pathway activation by reducing IL-6, IL-1β, and TNF-α protein/mRNA expression, suppressing iNOS and cyclooxygenase-2 (COX-2) enzymes and their downstream products (NO and prostaglandin E2 (PGE2)), blocking p38 and c-Jun N-terminal kinase (JNK) phosphorylation, and inhibiting NF-κB p65 nuclear translocation, thereby validating its regulatory role in TLR4 downstream NF-κB/MAPK pathways (102). I. Further studies using LPS/ATP co-activated RAW264.7 cell models revealed that CGA suppresses inflammasome activation via miR-155-mediated epigenetic regulation. Specifically, CGA dose-dependently downregulated NLRP3 expression and caspase-1 activity, inhibited IL-1β/IL-18 maturation and release, and blocked NF-κB nuclear translocation while concurrently reducing microRNA-155 (miR-155) levels. Functional rescue experiments confirmed that miR-155 inhibitors replicated CGA's anti-inflammatory effects, whereas miR-155 mimics reverse these actions, elucidating CGA's cascade inhibitory effects through the miR-155/NF-κB/NLRP3 signaling axis (103).

#### 3.3.3 Cafestol and Kahweol

Studies have demonstrated the therapeutic potential of kahweol and cafestol across multiple inflammatory models (Figure 4). Kahweol significantly reduces the expression of cytokines such as IL-6 and TNF-α, and chemokine (CXCL8) in human keratinocyte HaCaT cells by suppressing MAPK, NF-κB, and signal transducer and activator of transcription (STAT) signaling cascades (104). In hepatic inflammation models, Kahweol effectively alleviates liver inflammatory damage through downregulation of NF-κB and STAT3 activation in primary Kupffer cells and hepatocytes (105). Further investigations revealed that kahweol suppresses IL-2 secretion in phytohemagglutinin (PHA)/phorbol myristate acetate (PMA)-activated Jurkat lymphocytes via ERK/c-Fos signaling axis blockade, further confirming its anti-inflammatory activity (106).

### 3.4 Antioxidant

Oxidative stress refers to a pathological imbalance wherein the production rate of ROS exceeds the clearance capacity of the antioxidant defense system (107, 108). Under normal physiological conditions, ROS generated via the mitochondrial electron transport chain, NADPH oxidase, and other metabolic pathways—such as superoxide anion (O_2_·−) and hydrogen peroxide (H_2_O_2_)—participate in cellular signal transduction and immune regulation. When exogenous stimuli (e.g., ultraviolet radiation, environmental pollutants) or endogenous metabolic disorders lead to excessive ROS accumulation, the compensatory capacity of antioxidant enzyme systems—including SOD, CAT, and GSH—is overwhelmed, triggering lipid peroxidation (reflected by elevated malondialdehyde levels), protein carbonylation (increased protein carbonyl content), and DNA oxidative damage (107, 108). This disruption of redox homeostasis activates pro-inflammatory signaling pathways such as NF-κB/NLRP3, inducing programmed cell death and contributing to the pathogenesis of major diseases including neurodegenerative disorders, atherosclerosis, and malignancies. Consequently, restoring the oxidant-antioxidant dynamic equilibrium has emerged as a critical therapeutic strategy for modulating disease progression (107).

#### 3.4.1 Caffeine

**Figure 5:**
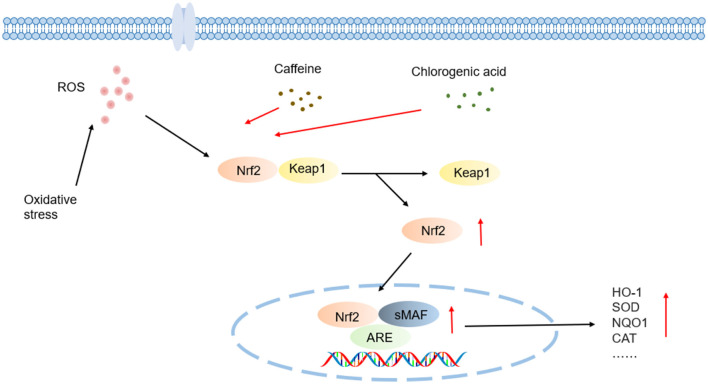
Schematic illustration of antioxidant effects mechanisms underlying coffee's core functional components.

Caffeine alleviates oxidative stress damage by activating the Nrf2/ARE pathway to enhance the expression of endogenous antioxidant enzymes such as SOD and CAT (Figure 5). In a hyaluronic acid-induced ocular hypertension rat model, caffeine significantly suppressed ocular oxidative stress—evidenced by reduced MDA levels and elevated SOD activity—effectively protecting visual pathway neurons from oxidative damage, despite having no intraocular pressure-lowering effect was observed (107). In hyperoxia-induced bronchopulmonary dysplasia (BPD) models using neonatal rats, caffeine restored oxidative balance by normalizing lipid peroxidation to normoxic levels, upregulating Nrf2 expression, and inhibiting its negative regulator Kelch-like ECH-associated protein 1 (Keap1), thereby reinstating SOD1 antioxidant function (108).

Studies employing oxalate-treated Madin-Darby canine kidney (MDCK) epithelial cells demonstrated caffeine pretreatment inhibits oxidized protein accumulation via restoring Nrf2 pathway activity and downregulating the Snail1 transcription factor. Nrf2 gene silencing experiments confirmed this pathway as caffeine's core mechanism against epithelial-mesenchymal transition (EMT) (109). In models of hyperoxia-damaged type II alveolar epithelial cells (AEC II), caffeine suppressed ERK1/2 phosphorylation, apoptosis, and proliferation inhibition by antagonizing A_2A_ receptors and disrupting the downstream cAMP/PKA/Src signaling cascade. A_2A_ receptor knockout experiments further validated this pathway's central regulatory role, while A_2A_ receptor agonists partially counteracted caffeine's protective effects (110).

#### 3.4.2 Chlorogenic acid

Chlorogenic acid (CGA) exerts synergistic antioxidant effects through direct scavenge of free radicals such as ·OH, O_2_·−, activation of the Nrf2/Keap1-ARE pathway, and upregulation of antioxidant enzymes such as HO-1, SOD, and CAT (Figure 5). The catechol moiety of 3,4-dihydroxybenzoic acid unit in its caffeoyl group interacts specifically with free radicals via a hydrogen-donating mechanism: during radical quenching process, adjacent phenolic hydroxyl groups transfer protons to highly reactive radicals like superoxide anion (O_2_·−) and hydroxyl radical (·OH) through hydrogen atom transfer (HAT) or single electron transfer (SET) reactions, forming thermodynamically stable semiquinone radical intermediates. These intermediates, stabilized by an intramolecular conjugated system through electron delocalization, effectively block radical-mediated chain oxidation reactions. This electron delocalization significantly lowers the redox potential of phenoxyl radicals and enhances the kinetic stability of antioxidant reactions (111).

In H_2_O_2_-induced oxidative damage models using PC12 cells, CGA demonstrated significant cytoprotective effects by lowering intracellular ROS levels, reducing lactate dehydrogenase LDH release and decreasing apoptosis rates, while activating the Nrf2 signaling axis to induce phase II detoxifying enzymes—including HO-1, NAD(P)H:quinone oxidoreductase 1 (NQO1), GSH, thioredoxin reductase 1 (TrxR1), and thioredoxin 1 (Trx1). Nrf2 gene silencing experiments confirmed this transcription factor as the pivotal regulator of CGA's neuroprotective effects (112). Additionally, in doxorubicin (DOX)-induced cardiotoxicity rat models, CGA significantly suppressed oxidative stress damage and cardiomyocyte programmed death through Nrf2/HO-1 pathway modulation, further validating its multi-target antioxidant mechanisms (113).

## 4 Concluding remarks and prospects

As a complex system spanning cultural, scientific, and commercial domains, coffee health research has evolved from early “risk assessment” paradigms into a new era of “precision nutrition.” Investigating the health effects of coffee fundamentally represents a scientific exploration interrogating the interplay between its inherent natural chemical complexity and human biological diversity. This review systematically elucidates the chemobiological properties of key bioactive components in coffee—including polyphenols, alkaloids, and diterpenoids—and constructs molecular networks illustrating their multi-target mechanisms in regulating neuroprotection, metabolic homeostasis, oxidative stress, and inflammatory balance. Accumulating evidence repositions coffee not merely a “caffeine delivery vehicle,” but as a dynamic, biosynergistic system with diverse physiological benefits.

Current research paradigms encounter three critical limitations. First, the predominant focus on isolated components and linear pathway associations fails to replicate the synergistic/antagonistic interactions of multi-component systems under physiological consumption conditions. This underscores the need for systematic pharmacology approaches to unravel the compositional interplay of coffee. Second, the biological functions of minor constituents remain largely underexplored due to a disproportionate emphasis on caffeine and CGAs leaves, demanding functional omics technologies to unveil their regulatory potential of these lesser-known compounds. Third, an overreliance on *in vitro* experiments and rodent models constrains clinical translatability. To bridge this gap, an integrated validation framework encompassing *in vitro*, animal, and human studies is essential to advance coffee's transformation from an empirical consumed beverage to a precision-targeted nutritional intervention.
